# Alignment across taxonomic levels in strategies rather than in traits along elevational gradients

**DOI:** 10.1093/evlett/qraf023

**Published:** 2025-08-26

**Authors:** Aaditya Narasimhan, Yvonne Willi

**Affiliations:** Department of Environmental Sciences, University of Basel, Basel, Switzerland; Department of Environmental Sciences, University of Basel, Basel, Switzerland

**Keywords:** elevational gradients, divergent adaptation, life-history strategies, micro- and macroevolution, parallelism, trait clines

## Abstract

Trait variation along environmental gradients can indicate the different strategies that organisms have evolved in response to environmental heterogeneity. So far, many inferences on trait–environment associations come from global studies performed at high taxonomic levels, and their transferability to lower evolutionary and spatial scales is unclear. Here, we tested for alignment in trait–environment relationships in 13 life-history and physiological traits among and within 7 Brassicaceae species over elevational gradients. Species and source populations originated from different elevations in the central Alps, and plants were raised under benign and warm conditions in greenhouse chambers. Some traits showed alignment in both within- and among-species trait clines, with even higher alignment on the level of trait complexes. There was high parallelism in resource allocation to leaves, particularly of carbon, with allocation decreasing with elevation. Size and biomass also decreased with elevation at the species and within-species level, respectively. Overall, the concordance in resource investment strategies when coping with lower as compared to higher elevations across evolutionary and spatial scales highlights their general role in adaptation to elevation.

## Introduction

The distribution of variation in life history and functional traits along environmental gradients is a central theme in evolutionary ecology. Insights allow the inference of optimal strategies favored under particular environmental conditions (Grime, 1977; McGill et al., 2006; Reich, 2014), as well as the roles of traits in adaptive divergence (Halbritter et al., 2018). The scale at which organisms are studied bears significance, as trait association approaches (Westoby, 2025) can be applied above or below the level of the species. This dichotomy brings forth a central issue in biology, whether patterns align on these two taxonomic levels (Rolland et al., 2023), and more generally, the extent to which evolution is parallel, i.e., whether similar trait changes occur along environmental gradients among taxa due to shared ancestry (Simpson, 1961). The search for parallelism has attracted increasing attention, as it would increase predictability of evolutionary responses to environmental challenges (Urban et al., 2016, 2023).

**Figure 1.**
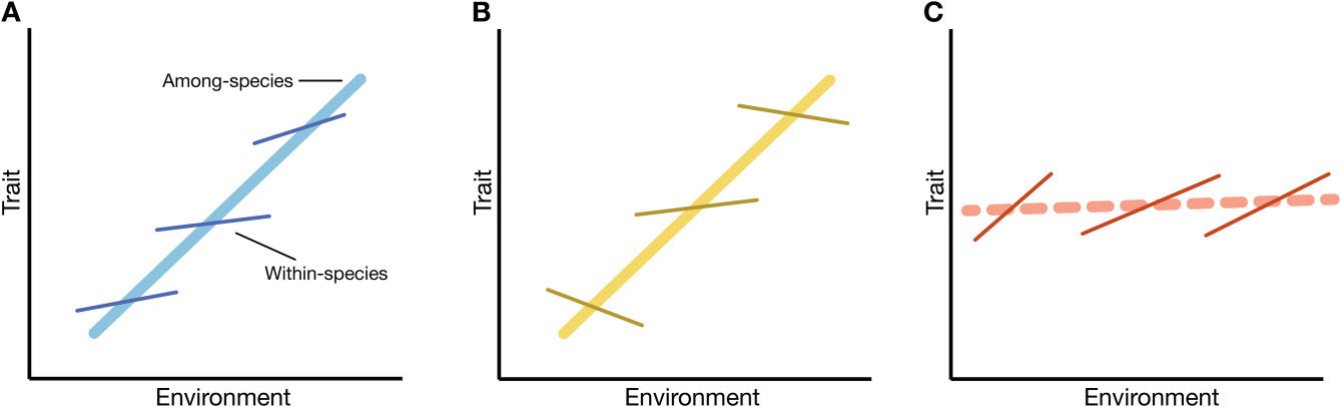
Evolved trait–environment relationships among related species (thick lines with brighter colors) and among populations within species (thin lines with darker colors, each representing a species) may generally align (A), or not, with either population clines being absent or inconsistent (B), or species clines being absent (C). The first scenario suggests consistency in selection on traits with shared trait architecture. The latter two scenarios may stem from selection differing over spatial scales or evolutionary times (B and C), gene flow among populations, or divergence in trait architecture weakening relations (B).

The extent to which the shape of a genetically controlled trait–environment association based on related species is similar to that on populations within species depends on several factors. Alignment between among- and within-species associations estimated in a common environment (Figure 1A) suggests that selection has operated in a similar manner on the same trait(s), resulting in parallel adaptation (Simpson, 1961; Wood et al., 2005). Parallel trait change is more likely if trait variation has a shared genetic and developmental basis among lineages, possibly coupled with an evolutionary legacy effect (Cavender-Bares et al., 2016; Losos, 2010). A lack of alignment at the two levels of grouping—resembling Simpson's paradox in statistical analysis—may have various causes. Inconsistent associations within but not among species (Figure 1B) may be due to gene flow among populations (Lenormand, 2002) or a nonconstant change in selection over the gradient (Geroldinger & Bürger, 2015; Hadfield, 2016). A scenario of strong within-species clines but none among species (Figure 1C) may arise for traits more strongly influenced by contemporary environmental conditions compared to long-term environmental effects (Famiglietti et al., 2024). Overall, consistent divergent selection over space and time on traits relevant to adaptation should generally lead to aligning patterns across taxonomic levels.

Studies that aggregate trait data to relate them to environmental gradients such as temperature or aridity often work on large spatial and phylogenetic scales, with seemingly little overlap with patterns at lower taxonomic or spatial levels. For example, a crucial trait of drought tolerance in land plants, resistance to embolism in vessels, exhibited weak correlations with climate when species within gymnosperms and angiosperms were analyzed (Choat et al., 2012). However, a strong aridity–embolism resistance relationship was observed in Cupressaceae, most likely due to a legacy effect of sharing only one of two common drought adaptation pathways (Brodribb et al., 2014). However, close relatedness may not always lead to alignment. In the radiation of the genus *Pelargonium* in South Africa, clines in leaf traits along climate gradients were mostly unique to subclades despite the recent shared ancestry of the taxa involved (Moore et al., 2018). In general, our current understanding of the relationships between traits and the environment, particularly the adaptive strategies of plants to climate variability, is largely derived from global studies on easily measurable traits. However, as illustrated by the above examples, alignment and/or parallelism may often be absent across taxonomic and spatial scales. One reason for this may be an insufficient mechanistic knowledge of trait functioning in a particular environment and various confounding factors that may be involved in trait–environment relationships (Anderegg, 2023).

Elevational gradients provide a “natural experiment” for investigating ecological and evolutionary changes when a small number of abiotic factors change predictably, notably temperature (Körner, 2007). A meta-analysis by Read et al. (2014) on functional trait–elevation relationships in plants revealed convergent effects on some traits at the species level. Leaf mass per area decreased with elevation; however, there was no relationship at the within-species level. Leaf nitrogen content (N_mass_), another trait typically associated with photosynthetic activity, showed no relationship with elevation at any taxonomic level, and within-species trait–elevation relationships generally exhibited idiosyncratic patterns. Furthermore, studies using experimental approaches suggested a genetic basis for variation along elevational gradients. In general, studies investigating trait–environment relationships over elevational gradients have rarely found convergent patterns across taxonomic levels (Cruz-Maldonado et al., 2021; Kichenin et al., 2013; Midolo et al., 2019; Mitchell et al., 2015; Moore et al., 2018; Weemstra et al., 2022). Also, a limitation of such studies is that they may insufficiently sample within-species variation or were based on field estimates and therefore unable to differentiate between presumably adaptive differences and immediate environmental effects (Anderegg, 2023).

**Figure 2.**
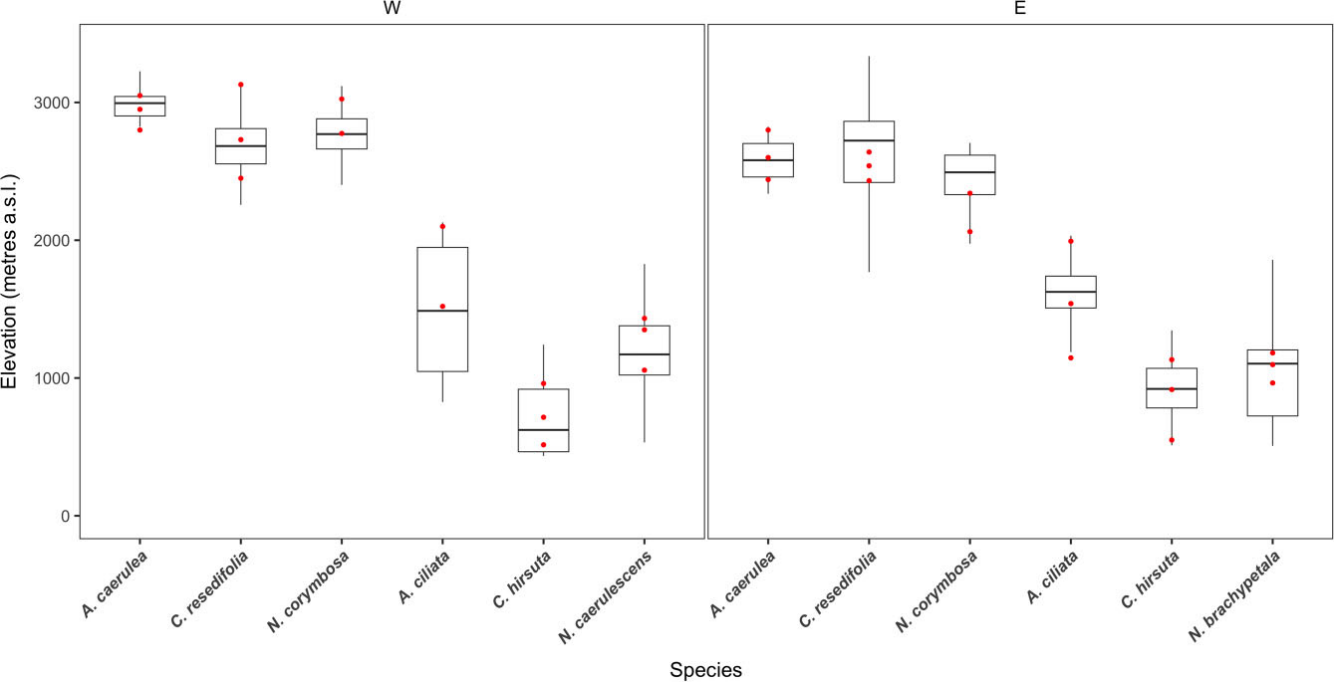
Elevation of sampled populations of the seven study species. For each species, we sampled seeds on two mountain transects, one in the western (W) part of Switzerland, on the left, and one in the eastern (E), on the right. On each transect, populations were sampled at three elevations: low, middle, and high (dots). In each panel, the first three species are from high elevations, followed by three low-elevation species. The boxplots show the local occurrences within a region with 5 km radius from the sampling transect. For *N. brachypetala* and *N. caerulescens*, the region was extended to include all of Switzerland. For *C. hirsuta* and *C. resedifolia* in the western and eastern regions, respectively, the local region was extended to 30 km radius. These were extended due to few or no reported occurrences in the local region. Occurrence data were obtained from *infoflora.ch*.

Here, we examined trait variation among and within species along elevational gradients under common garden settings using seven herbaceous plant species from the Brassicaceae family. Together, the selected species span wide elevational ranges across Switzerland, from montane to alpine environments. For each species, we sampled seeds of populations at two elevational transects of different biogeographic regions, with a population from the lower, middle, and upper end of each transect, ensuring range-wide sampling over elevation (Figure 2). Field-collected seeds were first propagated in the greenhouse for one generation, followed by raising offspring in common garden conditions for trait measurements. For Brassicaceae in the central Alps, high- and low-elevation species (alpine and montane, respectively) are divergent mainly in aspects of growth, acquisition, and allocation (Maccagni & Willi, 2022). The authors found that across growing conditions, high-elevation species had smaller leaves, and their leaf dry matter content (LDMC) was lower. Furthermore, growth under warm conditions induced changes in the expression of traits that differed among high- and low-elevation species. These findings motivated us to raise, measure, and compare populations and species under both control and warmer conditions to draw robust conclusions about genetically based trait–elevation relationships. Our main objective was to find the extent to which trait–environment relationships found on a microevolutionary scale, within species, aligned with those on a macroevolutionary scale, among species.

## Material and methods

### Study system

We selected seven species from three genera of the Brassicaceae family—*Arabis, Cardamine*, and *Noccaea*. Four were montane species (*Arabis ciliata, Cardamine hirsuta, Noccaea brachypetala*, and *N. caerulescens*) and the others were alpine, growing above the treeline (*A. caerualea, C. resedifolia*, and *N. corymbosa*). All species are diploid and predominantly selfing (Hay et al., 2014; Kimata, 1983; Lihová et al., 2009; Sterckeman et al., 2019), except for *Noccaea corymbosa*, which is a self-incompatible and outcrossing species. For each species, populations were sampled on two elevational transects, each on a different mountain slope (or area), in different main watersheds of Switzerland. For the two montane *Noccaea*, we sampled only one transect each because they were previously described as subspecies of *Thlaspi alpestre* (Koch & German, 2013) and are more closely related than other species of the same genus. On each transect, we sampled seeds (in 2021–2022) from a low, a central, and a high elevation population, often the lowest and highest known. Therefore, our sampling covered a large portion of the elevational range within the local region (e.g., a 5-km radius) of the sampling sites (Figure 2). For *N. corymbosa*, we could only sample two populations per transect, and in the “western” transect of *A. ciliata*, we also had only two populations (details in Table S1).

At each sampling site, seeds of 50 mother plants were collected over an area of 200 m^2^. We ensured each maternal plant was 1–2 m apart, to avoid sampling close relatives or clones. Field-collected seeds of each mother plant were stored separately under dry, dark, and cool conditions. Seeds were sown and propagated in the greenhouse for one generation to reduce the influence of the environment of origin (Kawecki et al., 2012; Rose, 1984 ). All except one species, *N. corymbosa*, were propagated by autonomous self-pollination. Plants of *N. corymbosa* were difficult to cross in the greenhouse, so we used field-collected seeds for the experiment.

### Plant rearing for the experiment

We raised four replicate plants from each of five randomly selected seed families per population under control and under warm conditions. In total, we targeted for 7 species × 3–6 populations × 5 seed families × 2 environments × 4 replicates = 1,320 plants, split over four spatial blocks in each treatment. The total number of plants raised was 1,224, due to lower germination rates of some species/populations/families. On average, each species–population–treatment combination had more than 18 plants (range 10–24, mean = 18.5, and target = 20). Due to logistical limitations, we performed the experiment across two rounds of sowing—both *Arabis* species first and the rest of the species in a second round.

Prior to sowing, seeds of *C. resedifolia* and the three *Noccaea* species were treated with 0.5 ml gibberellic acid (500 ppm, Merck KGeA, Dornstadt, Germany) to increase germination rates (based on observations from the propagation phase). Treated seeds were stored in the dark at 4 °C for 1 week (2 weeks for *N. corymbosa*). For the rest of the species, germination rates reflected their natural propensity to germinate. Untreated seeds were sown directly in multipot trays (0.06 L, gvz-rossat.ch, Otelfingen, Switzerland) containing a 1:2 mixture of sand (0–4 mm) and peat (Tref Go PP7 Substrate, gvz-rossat.ch, Otelfingen, Switzerland), and then stratified at 4 °C in the dark for 14 days in climate chambers (ClimeCabs, CLF Plant Climatics, Wertingen, Germany). Seeds treated with gibberellic acid were not stratified. Pots were watered regularly with tap water. During the germination phase, trays were covered with perforated plastic nets and kept at 18 °C with 75%–80% humidity and natural light in the greenhouse (∼9 hr of light). Pots were kept wet by spraying them from above and watering from underneath every 2–3 days. The nets were removed when >60% of pots of a tray had a germinated seedling, and intense watering was stopped once >75% of seeds in a tray had germinated. We thinned pots to one plant per pot when >75% of pots had large enough seedlings (usually around 2–3 weeks post sowing/removal from stratification). Excess seedlings were used to fill empty pots if a particular seed family was lacking enough replicates and shifted plants into their respective treatments. Further details on plant rearing are provided in the supplementary materials.

#### Treatments

The two conditions were *control*: 20 °C from 08:00–18:00, 18 °C at night, and *warm*: 20 °C (beginning of day), rising gradually to 30 °C for 2 hr (12:00–14:00), and gradually back to 20 °C, with 20 °C at night. Based on growing season records in the field (Körner, 2021), alpine plants can often experience up to 45 °C. For a greenhouse experiment, such high temperatures were unfeasible, and we decided to opt for a lower temperature peak of 30 °C, whereas the control treatment had a common standard temperature. Our warm treatment was not designed to heavily stress the plants but instead provided a moderate warming effect (average temperature in the warm treatment was 4 K higher; Figure S1). We reared plants across four greenhouse chambers, with two chambers for each treatment, each containing two spatial blocks. Much of the heterogeneity in the greenhouse chambers was within a chamber due to varying light intensities (150–250 μmol m^−2^ s^−1^), so we randomized trays within blocks within a chamber twice per week and between chambers once per week. Daylength was progressively increased every 3–4 days until the plants experienced 16-hr days. We watered whole trays every 2–3 days, slightly more frequently in the warm treatment to prevent any effects of drought. Plants were kept in their respective treatments until the first plants started bolting, when trait assessments were performed.

#### Trait measurements

Before treatments began, the time to germination (in days since end of stratification or end of gibberellic acid treatment) was assessed by checking every day whether the cotyledons had opened completely. Growth was measured by scanning plants twice a week using a 3D image scanner (Phenospex PlantEye F6000, Heerlen, The Netherlands) from the start of treatment until 2 weeks after leaf trait measurements. We measured “3D leaf area,” which captured the live leaf area, or the rosette size, in a plant and estimated growth trajectories from this trait (Figure S2). A three-parameter logistic-growth model best described our data (Figures S3 and S4), with the parameters of asymptotic rosette size (asym, in mm^2^), time to reach the mid-point of final size (*x*_mid_, in days), and maximal growth rate (*r*). We measured the following leaf functional traits: leaf area (LA, in mm^2^), specific leaf area (SLA, LA/dry weight, in mm^2^ mg^−1^), and LDMC (ratio of dry weight over fresh weight, in mg g^−1^). By stable isotope analysis, we estimated water-use efficiency using *δ*^13^C isotopic discrimination (Farquhar et al., 1989) (WUE, in ‰), leaf bulk carbon (C_mass_), and leaf nitrogen content (N_mass_) (isotope analysis performed by the Cornell University Stable Isotope Laboratory, Ithaca, NY, USA). Water-use efficiency (using isotopic discrimination) was calculated as 1,000 × (−0.0085–*δ*^13^C/1,000)/(1 + *δ*^13^C/1,000) (Seibt et al., 2008), where −8.5‰ was used as the *δ*^13^C value for air. A higher value of *δ*^13^C in C3 plants (i.e., those in the present study) indicates that the stomata are more often closed to conserve water and therefore the photosynthesis machinery discriminates less against ^13^C. For WUE, N_mass_, and C_mass_, we only sampled up to three replicates per seed family.

Following these trait assessments, we let plants flower under their respective growth treatment (those that required vernalization—all *Noccaea* species and *A. ciliata*—were shifted to growth chambers and kept under 4 °C with 8 hr of light for 5 weeks). Approximately 1 month after cumulative flowering reached a plateau, we harvested plants to measure above- and belowground dry biomass. Our analyses include 13 traits, 4 related to size (asym, LA, total biomass, aboveground biomass), 2 related to the speed of growth (*x*_mid_, *r*), 3 related to photosynthetic/acquisition capacity (N_mass_, SLA, WUE), and 4 related to allocation of carbon and resources (LDMC, root:shoot ratio, below-ground biomass, C_mass_). More details on trait assessments are in the supplementary materials.

### Statistical analysis

All traits were corrected for tray and block for each species–treatment combination separately prior to analysis (Supplementary document, section S4). Next, we standardized all traits using the grand mean and standard deviation across all species and treatments to improve model convergence. All traits were analyzed with multilevel models fit within a Bayesian framework utilizing a Hamiltonian Monte–Carlo algorithm with the R package “brms” (Bürkner, 2017). Each trait was modeled with the fixed effects of deviation of each population from the species’ median elevation, median elevation of species’ distribution in Switzerland (based on occurrence reports on *infoflora.ch*), treatment, and the two-way interactions involving elevations with treatment. Parametrizing elevation into a within-species component (deviation from the median) and an among-species component (median species’ elevation) allowed the disentangling of within- and among-species trait–elevation relationships (Liu et al., 2023). We accounted for species relatedness, varying intercepts, and (uncorrelated) varying slopes for deviation from median elevation at the level of species, and varying intercepts both for population within species and for the seed family in each population-species combination. Such a variance structure allowed us to partition variance among species in the within-species trait–elevation relationships. The phylogeny was obtained by pruning a recently published phylogeny based on several chloroplast genes (Patsiou et al., 2021), and a relatedness matrix was generated using the R packages “ape,” “geiger,” and “phytools” (Paradis & Schliep, 2018; Pennell et al., 2014; Revell, 2024).

As multiple factors were included in our model (e.g., species, populations, treatments), not all traits satisfied the assumption of equal variances among groups or approximated a Gaussian distribution. However, model fitting in “brms” is flexible; it allows accounting for heterogeneity in variances by modeling the scale (standard deviation) parameter separately, or including skewed distributions. Details on model setup for each trait are provided in the supplementary materials. All models were run for enough iterations to achieve an effective sample size of at least 1,000 for each estimated parameter. Convergence was checked using Rhat values and visually inspecting the MCMC (Markov chain Monte Carlo) chains. Posterior predictive checks were further used to verify whether the models fit the data. The R package “marginaleffects” (Arel-Bundock et al., 2024) was used for estimating effects and “tidybayes” (Kay, 2024) for visualization.

For presenting and discussing results, we considered four functional trait complexes (Table 1): size, speed of growth, acquisition, and allocation to above- and belowground organs. These trait complexes involve important aspects of a plant's ontogeny, structure, and function (Laurans et al., 2024).

**Table 1.** Marginal effect estimates, i.e., slopes, of elevation among and within species, and the growth treatment.

Traits	Elevation: among species		Elevation: within species		Treatment
	Control	Warm	Control	Warm	Warm vs. control
**Size**					
Asymptotic size	−0.196 (−0.842, 0.474)	−0.302 (−0.974, 0.344)	0.152 (−0.150, 0.435)	−0.180 (−0.466, 0.112)	**−0.138 (−0.209, −0.073)**
Leaf area	**−0.770 (−1.023, −0.479)**	**−0.857 (−1.124, −0.584)**	0.006 (−0.282, 0.318)	0.032 (−0.266, 0.334)	***-0.067 (−0.113, −0.021)***
Total biomass	−0.217 (−0.767, 0.347)	−0.294 (−0.871, 0.242)	**−0.236 (−0.449, −0.007)**	**−0.406 (−0.627, −0.183)**	***0.054 (0.005, 0.096)***
Aboveground biomass	−0.167 (−0.753, 0.375)	−0.248 (−0.815, 0.320)	**−0.291 (−0.534, −0.073)**	**−0.362 (−0.599, −0.144)**	***0.084 (0.044, 0.128)***
**Speed of growth**					
Time to fastest growth	0.358 (−0.377, 1.064)	0.455 (−0.296, 1.145)	0.182 (−0.103, 0.446)	−0.120 (−0.392, 0.154)	***0.074 (0.026, 0.124)***
Growth rate	−0.088 (−0.390, 0.187)	−0.014 (−0.289, 0.291)	0.048 (−0.061, 0.157)	0.116 (−0.001, 0.224)	***-0.037 (−0.065, −0.011)***
**Acquisition**					
Leaf nitrogen content	−0.033 (−0.502, 0.390)	−0.251 (−0.730, 0.172)	0.257 (−0.136, 0.649)	0.150 (−0.254, 0.545)	**0.254 (0.174, 0.328)**
Specific leaf area	−0.257 (−0.857, 0.397)	−0.439 (−1.047, 0.213)	0.404 (−0.035, 0.799)	0.232 (−0.199, 0.631)	**0.232 (0.168, 0.297)**
Water-use efficiency	0.342 (−0.291, 0.948)	0.348 (−0.261, 0.994)	0.033 (−0.237, 0.324)	−0.079 (−0.357, 0.200)	**0.100 (0.042, 0.156)**
**Allocation**					
Leaf dry matter content	**−0.647 (−0.958, −0.326)**	**−0.679 (−0.980, −0.35)**	**−0.519 (−0.895, −0.151)**	−0.129 (−0.523, 0.238)	**−0.202 (−0.264, −0.144)**
Root:shoot ratio	0.223 (−0.450, 0.918)	0.051 (−0.667, 0.704)	**0.598 (0.162, 1.011)**	0.359 (−0.088, 0.758)	**−0.176 (−0.214, −0.141)**
Belowground biomass	−0.375 (−1.102, 0.390)	−0.472 (−1.167, 0.328)	0.189 (−0.044, 0.404)	**−0.383 (−0.604, −0.157)**	**−0.126 (−0.179, −0.073)**
Leaf carbon content	**−0.400 (−0.665, −0.166)**	**−0.524 (−0.776, −0.272)**	**−0.368 (−0.641, −0.112)**	**−0.422 (−0.688, −0.169)**	**−0.160 (−0.228, −0.101)**

*Note*. All models were fit using Bayesian multilevel models. Effects (medians) and their 90% highest posterior density (HPD) intervals are reported, with estimates in bold having intervals that do not include zero, and in italics estimates <|0.1|. Varying slopes and intercepts on the level of random effects are not shown. Prior to analysis and plotting, traits were rescaled to a mean of 0 and a standard deviation of 1 across all species and treatments.

## Results

**Figure 3.**
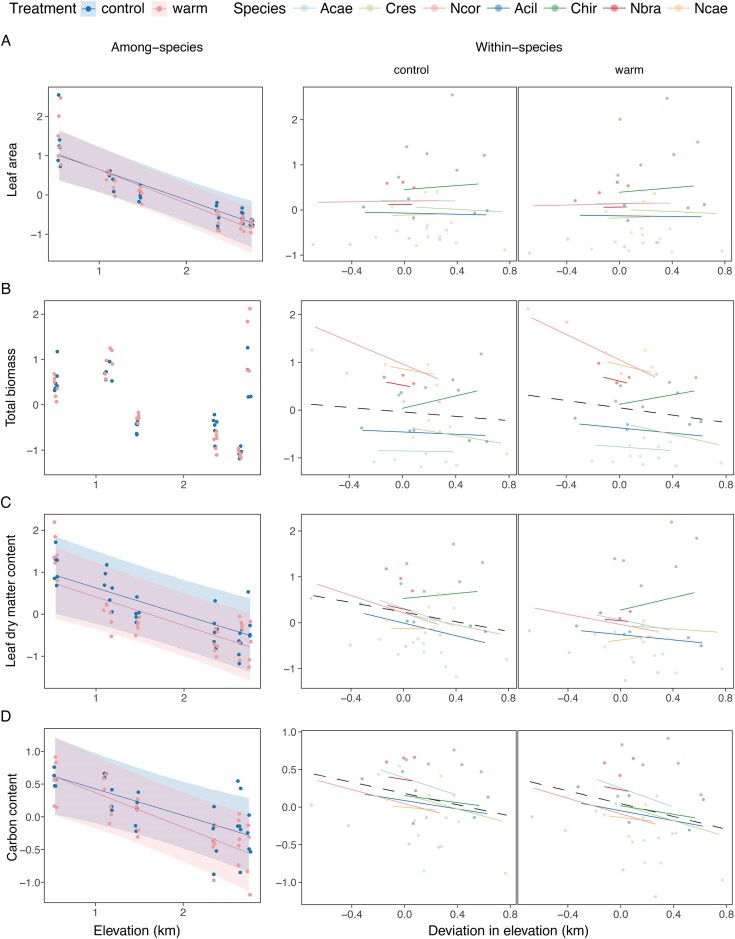
Trait–elevation relationships for size and allocation traits, (A) leaf area (LA), (B) total biomass, (C) leaf dry matter content (LDMC), and (D) leaf carbon content (C_mass_). Among- and within-species trait–elevation relationships are shown on the left and right panels, respectively. Lines indicate whether a general among-species or within-species (dashed lines) relationship was found. For panels on the left, shaded regions around the slopes depict 90% highest density continuous intervals (HDCI). Panels on the right depict within-species patterns, and slopes for each species highlight the variance among within-species slopes. Points depict population-level means for each trait and were jittered slightly for better visibility. Prior to analysis and plotting, traits were rescaled to a mean of 0 and a standard deviation of 1 across all species and treatments.

Half of the examined traits exhibited correlations with elevation, either within or among species, or both. At the species level, significant trait–elevation relationships were observed for leaf area, LDMC, and carbon content (Table 1). Higher-elevation species had smaller leaves (Figure 3A, on the left) with reduced dry matter content and lower carbon content (Figure 3C and D). The slopes of these relationships were consistent across the two treatments.

For two of these traits, LDMC and carbon content, strong alignment was found across taxonomic levels (Table 1). Under control conditions, these traits exhibited negative relationships with elevation within species, and additionally, under warm conditions, carbon content also showed a negative correlation with elevation (Figure 3C and D, on the right).The strength of these trait–environment relationships was comparable between the among- and within-species levels. For instance, a within-species slope of ∼-0.5 for LDMC in the control treatment indicates that a 1000-m change in elevation corresponds to a decrease of ∼50% of the overall phenotypic standard deviation, reflecting a substantial change. Similarly, among-species LDMC decreased by 65% every 1,000 meters of elevation, also indicating a substantial change.

Additional traits exhibited associations with elevation within species. Specifically, total biomass and aboveground biomass decreased with elevation in both treatments (Figure 3B; Figure S6B). Furthermore, the root:shoot ratio increased with elevation, although only under control conditions (Figure S6C). This suggests a lower reduction of root biomass compared to shoot biomass with elevation. In the warm treatment, there was a reduction in root biomass, resulting in similar root:shoot ratios across the growth conditions (Figure S6C and D).

The warm treatment had a general effect on all plant traits measured. Acquisition traits exhibited more pronounced shifts, with leaf nitrogen content, SLA, and WUE increasing under warmer conditions (Table 1). In contrast, allocation traits such as LDMC, root:shoot ratio, and leaf carbon content were strongly reduced under warmer conditions. For size and speed of growth, the effects were weaker (with effect sizes less than 0.1 except for asymptotic size). These results align with phenotypes that adjust to warm phases by enhancing photosynthetic capacity and reducing allocation.

Variance among species in the within-species slope on elevation was low for most traits. An exception were the biomass traits with higher variances in within-species slopes (Figure S5), attributable to some species with idiosyncratic patterns (Figure 3B; Figure S6B). For LDMC under control conditions, most species had steep negative slopes, with two species, *N. caerulescens* and *C. hirsuta*, displaying weakened or opposite relationships, resulting in higher variance (Figure 3C). In the warm treatment, within-species slopes of LDMC on elevation were generally weaker, but still with considerable variance across species (Figure S5). Notably, C_mass_, which exhibited elevational trends similar to LDMC, displayed low variance in within-species patterns, indicating strong parallelism in elevational clines (Figure 3D; Figure S5). Within-species slopes for N_mass_ indicated weak relationships for most species, except for *N. corymbosa*, where N_mass_ exhibited a change of approximately half a standard deviation across its range (Figure S7).

## Discussion

The alignment of trait–environment associations across various taxonomic levels and the parallelism of within-species clines suggests a strong role for past and contemporary selection targeting the same traits, providing a high level of predictability in how traits respond to future environmental changes. In this study, we measured life-history and leaf functional traits known to diverge along elevation at a species level in Brassicaceae (Maccagni & Willi, 2022). Seven traits exhibited associations with elevation, but most—five—were not aligned across taxonomic levels. However, there was high concordance in the strategies they represented. Overall, we observed a decrease in size and allocation traits with elevation, but no changes in the speed of growth and acquisition capacity. We discuss our findings in the context of the alignment of micro- and macroevolutionary patterns and adaptation to elevation.

Leaf dry matter content and leaf carbon content displayed strong alignment across taxonomic levels (Figure 3C and D), suggesting both a historical and a contemporary role of selection toward reduced leaf materialization and relatively fewer carbon-rich structures with increasing elevation (scenario similar to Figure 1A). A role of a lower partial pressure of CO_2–_—resulting in reduced carbon uptake—at higher elevations is unlikely as high-elevation species are more efficient at carbon uptake and match low-elevation species in their photosynthetic gains per unit area (Körner, 2021). Low LDMC may also imply greater water-holding capacity in leaves, which may be favorable under drought and heat, given similar levels of WUE (Table 1). However, the strongest constraint faced by higher-elevation plants is actually a shorter growing season (Patsiou et al., 2021). The reduced resource investment particularly in carbon-rich structures may be indicative of a “fast” and more ephemeral life history (Pérez-Harguindeguy et al., 2013; Wilson et al., 1999), favored in places where the growing seasons are short.

For size traits, we found little alignment across taxonomic levels in individual traits, but consistency in the direction among size traits (Figure 3A and B; Figure S6 and Table 1). On an among-species level, leaf area varied with elevation, whereas on the within-species level, total biomass and aboveground biomass consistently declined with elevation. The finding of no decrease in LA along elevation within species is in line with other studies (Kichenin et al., 2013; Liu et al., 2023; Midolo et al., 2019). The near-zero association in LA within species (Figure 3A, resembling the scenario in Figure 1B), could be due to little divergent selection on LA across shorter elevational gradients within species, or the evolution of LA being constrained within species. The absence of clines in biomass-based size traits on the species level was unusual as biomass reduction with elevation is a common observation (Körner, 2021). In our study, the absence of any pattern was mainly due to the high-elevation *N. corymbosa*, which had unusually thick stems resulting in much higher biomass (Figure 3B). For within-species variation in belowground biomass, there were some nuances between treatments. Populations of higher elevations had higher root:shoot ratios under control conditions, but lower belowground biomass under warm conditions, indicative of plastic adjustments in resource allocation to roots in response to temperature (Table 1). Overall, size traits showed parallelism in clinal variation, but on the level of trait complexes.

Consistency in finding no trait clines within and among species for other aspects of life history and physiology also represents alignment—in their noninvolvement as strategies over the elevational gradient. The two traits related to the speed of growth and the three traits related to acquisition exhibited no associations with elevation, both at the level of species and populations within species. These results indicate that higher elevation does not select for faster vegetative development or a higher photosynthetic capacity in these alpine herbs, which may have been expected due to shorter growing seasons. The result is aligned with findings in a macroevolutionary study on 100 Brassicaceae species (Maccagni & Willi, 2022) but slightly differs as higher-elevation species showed a trend to speed up growth under warmer conditions (Maccagni & Willi, 2022). This difference is possibly due to the harsher thermal treatment in Maccagni and Willi (2022) with a peak of 40 °C as opposed to 30 °C in the present study, which can induce stress and trigger earlier growth.

The motivation to study trait–environment relationships stems from an assumption that the studied traits affect fitness (Violle et al., 2007). This need not always be the case, as some traits may be uninvolved or less involved in how they influence fitness depending on the environmental context (Shipley et al., 2016). Moreover, the environment may select for an “integrated phenotype” or “trait syndrome” representing a general strategy (Damián et al., 2020), which can be achieved via many redundant pathways (James et al., 2023). Our results suggest that pathways to elevational adaptation are complex, and plants can follow various paths (that need not strongly overlap). The general support for “integrated phenotypes” or “strategies” (groups of traits that achieve a similar functional output) suggests a less important role for specific “traits” and a more significant role for pathways to similar adaptive functions over elevational gradients. This is exemplified by size and allocation-based traits, which decreased with elevation among and/or within species, and may reflect a general adaptive strategy to elevation in Brassicaceae herbs. For example, *Arabidopsis arenosa* populations from foothill and alpine environments were locally adapted based on their size and survival. Although alpine individuals were smaller, they were larger than foothill plants transplanted to a high-elevation site and also had better survival, demonstrating that reduced allocation at higher elevations is an adaptive strategy (Wos et al., 2022). Our findings are also unlikely to be affected by important life-history differences between species, e.g., longevity. The alpine species we worked with were perennial, whereas *C. hirsuta* and low-elevation *Noccaea* were biennials, but there were no clear differences in results along this line.

In summary, our study highlights that limited alignment of macro- and microevolutionary trait–elevation relationships is common, given the myriad of processes that can contribute to nonalignment. We found no associations with elevation for half of the studied traits, indicating that perhaps they did not covary with fitness along elevation, though this nonetheless reflected alignment in their lack of association. There was a high degree of parallelism in the reduction of carbon allocation along elevation. Moreover, despite the limited alignment for individual traits, there was alignment in the strategies they represented, characterized by a reduction in size and leaf investment with elevation. Such a consistent pattern in a suite of related traits is indicative of their role in elevational adaptation and identifies a target set of traits for future studies. Although the generalizability of trait–environment relationships is difficult across contexts, our results are likely a general feature of herbaceous species distributed along elevational gradients in Central Europe. More studies that sample the extent of diversity within and among species should thus be encouraged, with traits measured in common gardens, to accurately identify the extent of alignment in trait–environment relationships and consequently infer adaptive strategies organisms use across various environments.

## Supplementary Material

qraf023_Supplemental_File

## Data Availability

All data and code required to reproduce the results in this paper have been deposited to Zenodo (https://doi.org/10.5281/zenodo.14677550).
