# Supplementary material for: Alignment across taxonomic levels in strategies rather than in traits along elevational gradients
Source: Evol Lett. 2025 Aug 26;9(5):567–75. doi: 10.1093/evlett/qraf023 (PMC12492251; doi:10.1093/evlett/qraf023)
Supplement: qraf023_Supplemental_File [file qraf023_supplemental_file.pdf]

Supplementary Material

Alignment across taxonomic levels in strategies but not in traits along elevational gradients

Aaditya Narasimhan* and Yvonne Willi

University of Basel, Switzerland

Email: aaditya.narasimhan@unibas.ch

Table of Contents

S1. Sampling details ***2***

S2. Methods: Plant rearing (additional information) ***3***

S3. Methods: Growth curve estimation ***4***

S4. Methods: Trait measurements ***9***

S5. Model setup (additional details) ***10***

S5.1 General terms **10**

S5.2 For LDMC, SLA, asym, WUE **11**

S5.3 For leaf carbon content and growth rate **11**

S5.4 Rest of the traits **11**

S6. Additional results ***13***

References ***16***

S1. Sampling details

Table S1: Table depicting the sampling locations and the source elevations. The species' median elevation represents the median elevational occurrence of the species across Switzerland.

Name	Elevation [m a.s.l]	Species elevation	Species median elevation [m a.s.l]	Transect position	Latitude [°N]	Longitude [°E]
Arabis caerulea	2440	high	2640	low	46.84	9.81
Arabis caerulea	2600	high	2640	mid	46.83	9.8
Arabis caerulea	2800	high	2640	high	46.84	9.8
Arabis caerulea	2800	high	2640	low	46.03	7.79
Arabis caerulea	2950	high	2640	mid	46.03	7.79
Arabis caerulea	3050	high	2640	high	46.02	7.8
Arabis ciliata	1146	low	1472	low	46.89	10.46
Arabis ciliata	1540	low	1472	mid	46.88	10.43
Arabis ciliata	1993	low	1472	high	46.81	10.27
Arabis ciliata	1520	low	1472	mid	46.33	7.69
Arabis ciliata	2100	low	1472	high	46.34	7.7
Cardamine hirsuta	550	low	542	low	47.07	8.59
Cardamine hirsuta	916	low	542	mid	46.28	7.4
Cardamine hirsuta	1133	low	542	high	47.07	8.59
Cardamine hirsuta	515	low	542	low	46.26	7.41
Cardamine hirsuta	715	low	542	mid	46.27	7.41
Cardamine hirsuta	960	low	542	high	46.28	7.4
Cardamine resedifolia	2432	high	2360	low	46.84	9.82
Cardamine resedifolia	2540	high	2360	mid	46.84	9.81
Cardamine resedifolia	2640	high	2360	high	46.83	9.8
Cardamine resedifolia	2450	high	2360	low	46	7.76
Cardamine resedifolia	2730	high	2360	mid	45.98	7.75
Cardamine resedifolia	3130	high	2360	high	45.98	7.79
Noccaea brachypetala	964	low	1104	low	46.92	7.93
Noccaea brachypetala	1097	low	1104	mid	46.92	7.92
Noccaea brachypetala	1182	low	1104	high	46.92	7.92
Noccaea caerulescens	1057	low	1171	low	47.1	6.94
Noccaea caerulescens	1350	low	1171	mid	47.09	6.9

Name	Elevation [m a.s.l]	Species elevation	Species median elevation [m a.s.l]	Transect position	Latitude [°N]	Longitude [°E]
Noccaea caerulescens	1433	low	1171	high	47.02	6.82
Noccaea corymbosa	2062	high	2748	low	46.33	8.85
Noccaea corymbosa	2341	high	2748	mid	46.3	9.07
Noccaea corymbosa	2775	high	2748	mid	45.98	7.76
Noccaea corymbosa	3025	high	2748	high	45.98	7.78

S2. Methods: Plant rearing (additional information)

Treatments – We reared plants across four greenhouse chambers, with two chambers for each treatment. Much of the heterogeneity in the greenhouse chambers was within a chamber (due to varying light intensities), so we randomised trays twice per week within a chamber and once per week between chambers. Daylength was progressively increased every 3-4 days until the plants experienced 16 hours of light. We watered whole trays every 2-3 days, slightly more frequently in the warm treatment, to prevent any effects of drought.

Plant protection – Trait assessment was done on fertilized plants. Around 7-10 days before leaf trait measurements and every 4 weeks thereafter, plants were fertilised (0.2% Wuxal, Westland Schweiz GmbH, Dielsdorf, Switzerland). When needed, plants were sprayed with pesticides for thrips once (0.02% Spintor, Biocontrol Andermatt, Switzerland) and aphids when present (Neudosan AF Neu, Neudorff, Germany).

A)

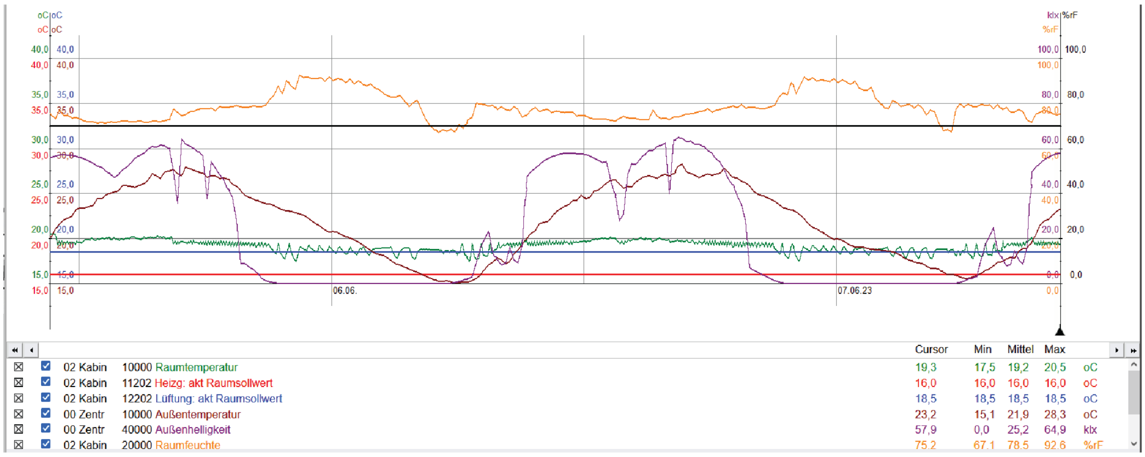

B)

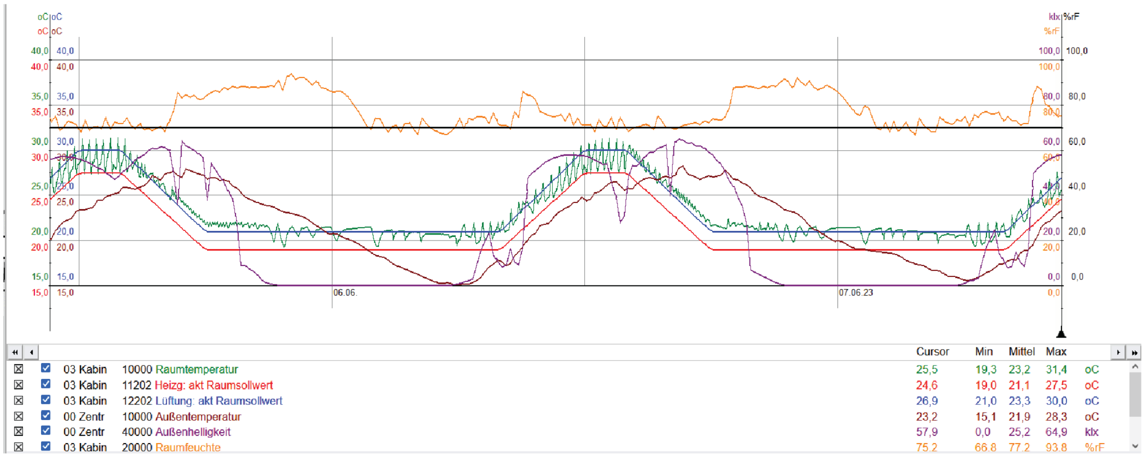

Figure S1: Greenhouse parameters for the control and warm treatments. A) In the control treatment, the average temperature over a day was 19.2°C, with a range of 17.5 – 20.5°C. B) In the warm treatment, the average temperature over a day was 23.2°C, with a range of 19.3 – 31.4°C. For both treatments, the green and yellow lines show the current room temperature and current humidity, respectively, with the x-axis depicting time and y-axis depicting either the temperature (left) or humidity (right).

S3. Methods: Growth curve estimation

We checked seed germination every day for the first 10 days and recorded this as the germination date. Plant growth was measured by scanning plants three times a week using a 3-D image scanner (Phenospex PlantEye F600, Heerlen, The Netherlands) from the start of treatment until the first plants started bolting, after which leaf trait measurements were performed. We also scanned plants two more times after this, once per week, to make the growth curve estimation process easier if needed. More information about the instrument and the associated software "HortControl" can be found on https://phenospex.com/products/plant-phenotyping/planteye-f600-multispectral-3d-scanner-for-plants/. Essentially, this device is composed of two scanners mounted on a rail that scans plants placed below it (Figure S2, left).

For each scan, we set filtering criteria to remove noise such as soil, mosses, and any non-plant material such as the pot and labels. These included restricting hue values between 0 and 177, NDVI (normalised difference vegetation index) between 0.35 and 1, and lightness between 6 and 100. The range of hue values represents greens along with some yellows and reds, encompassing the range of pigments seen on leaves. The NDVI filter removed "dead" material i.e., with values below 0.35. Finally, the lightness filter removed objects with low lightness that add to noise in the early phase of growth – soil and mosses.

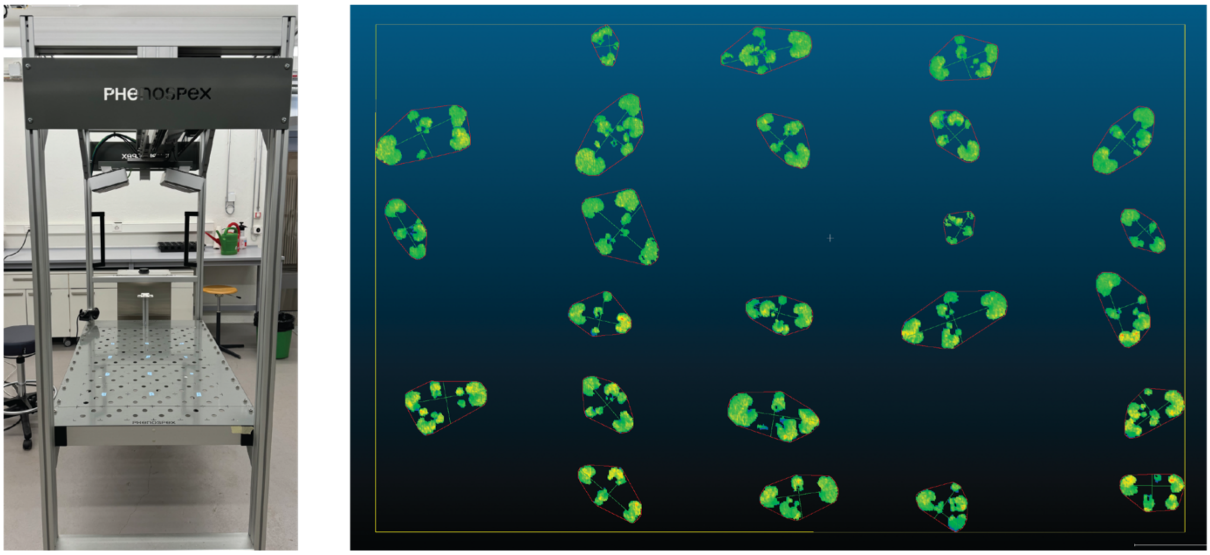

Figure S2: The Phenospex setup. Left. The two scanners can be seen on the top, mounted on a rail at an angle that ensures 3D scans. Right. An example of a 3D scan for *C. hirsuta*, during the earlier stages of growth.

We measured "3D leaf area" (Figure S2, right), which captured the live leaf area, or the rosette size, of a plant and estimated growth trajectories from this trait. The starting size for the day of germination was set to 2 mm^2, which was larger than seed sizes for all our species. We scanned plants three times a week, until the first plants started bolting. Apart from the first sowing round, we added two scans (one per week) after leaf trait measurements to ensure a plateau in case there were issues. We fit the plant size data to seven growth models: linear, exponential, power, 2- and 3-parameter logistic, Gompertz, and von Bertalanffy using the R package "minpack.lm" (Elzhov *et al.* 2023). Based on the weighted AIC values, we found the 2- and 3-parameter logistic models being the best, with the 3-parameter logistic model edging out as it was the better model for more species (Figures S3, S4). In general, all our rosette plants attained their final rosette size around the bolting phase, which further justified the use of a logistic model. The parameters described the growth trajectory, e.g., asymptotic rosette size

(asym, in mm^2), time taken to reach the mid-point of final size (xmid, days), and maximal growth rate (r).

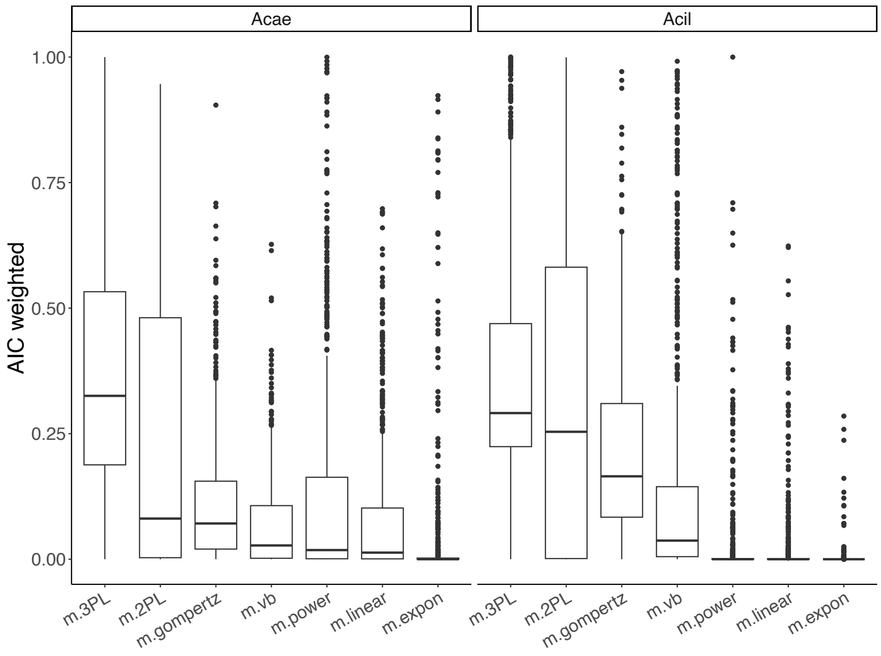

Figure S3: Boxplots of weighted AIC for the various growth models for *Arabis* species. The first four models are asymptotic ("m.3PL": 3-parameter logistic, "m.2PL": 2-parameter logistic, Gompertz, "m.vb": von Bertalanffy), and the next three have no asymptote (power, linear and exponential). The 3-parameter logistic model was overall the best supported across all species. Growth models were fit to unprocessed data, to select the most appropriate model, after which data were further processed to fit to the best model if required.

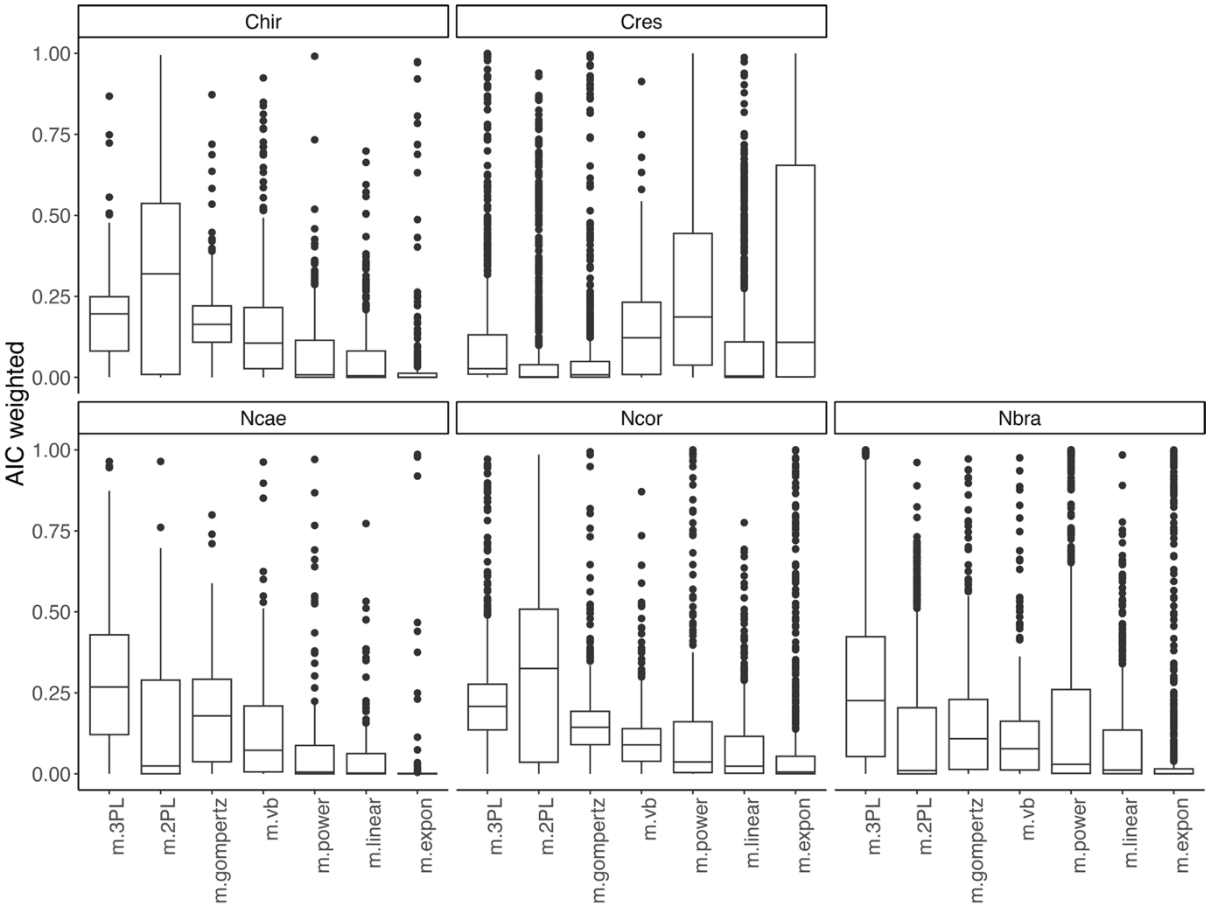

Figure S4: Boxplots of weighted AIC for the various growth models for *Cardamine* and *Noccaea* species. The first four models are asymptotic ("m.3PL": 3-parameter logistic, "m.2PL": 2-parameter logistic, Gompertz, "m.vb": von Bertalanffy), and the next three have no asymptote (power, linear and exponential). The 3-parameter logistic model was overall the best supported across all species. Growth models were fit to unprocessed data, to select the most appropriate model, after which data were further processed to fit to the best model if required.

Unfortunately, the package could not fit 3-parameter logistic (3.PL) curves for some plants due to noise or other reasons. In this case, we inspected the 3.PL fits visually for all the plants that were not fit, and devised solutions depending on the species:

1. *C. hirsuta*

- Round I: We had 61/762 plants with no fit, and this was due to some plants having significant (>20%) decreases in size and recovery in the next scan. We removed these unusually low values. Finally, we were left with 7/762 (<1%) plants with no fit.
- Round III: First, we removed the last two scans taken after leaf trait measurement (to make the number of scans similar across the two rounds). Then, we used the same

outlier removal procedure as described for Round I, which lowered the number of no fits from 12 to 2 (out of 267 plants), i.e., <1% had no fit in the end.

2. *Arabis* species

- Initially, we had 58/1597 plants with no fit. Removing the last two values after leaf trait measurement led to <1% non-fits (12/1597)

2. *C. resedifolia*

- The best fits were usually exponential, and size data did not allow the fitting of a 3-parameter logistic curve. Main reasons were the high leaf turnover in this species, and that after reaching an early plateau for rosette size, growth continued by filling in space within the rosette. Though, when 19 weeks after the last scan 116 plants were randomly selected and scanned again, rosette sizes were on average 17% smaller, supporting that a high size had been reached since the last round of scanning. Furthermore, we had evidence that the increase in size had started slowing (for 73% of plants) between the second last and last image taken after trait measuring. Both insights motivated us to use that last size estimate twice, in the week of attaining and 7 days later. With this data, we could fit 3-parameter logistic models for all but 5/904 plants.

3. *Noccaea brachypetala*

- Like *C. resedifolia*, for 57/923 plants, we had no fits. We added to the last size data the same estimates after 7 days. We also removed outliers using the same procedure as for *C. hirsuta*. Then, we were left with 3/923 non-fits (<1%).

4. *Noccaea corymbosa*

- For most plants, the growth trajectory resembled that of *C. resedifolia*. We added to the data the last estimate a second time 7 days after last image taking and removed outliers as in *C. hirsuta* to end up with 16/710 plants for which we were unable to fit the 3-parameter logistic growth model. These plants either did not grow after germination, or had stopped growing at some point, and therefore they were excluded from the experiment.

<u>Note</u>: The numbers reported above include all plants grown across all sowing rounds, some of which were not a part of this study. However, the percentage and numbers of “non-fits” are still provided for transparency. The final numbers for this study are listed below. The growth-curve-fitting procedure was adapted from Heblack et al. (2024).

Finally, all non-fits were assigned an asymptotic size equal to that of the maximum recorded size, with the growth rate and "xmid" values set to NA. To summarise, all species in the study reached a stable rosette size by the time the first plants started bolting, after which increases in rosette size did not occur. Therefore, our treatment of the few non-fits, to improve growth curve estimation, was an accurate representation of their growth. In the end, out of 1233 seeds that had germinated, we could fit growth curves to 1224 plants (99%), of which we measured leaf traits on 1196 (97.7%) plants. On average, each species-population-treatment combination had more than 18 plants (range 10-24, mean = 18.5, target = 20).

S4. Methods: Trait measurements

1. Growth traits: mentioned in section S03.
2. Leaf traits: This protocol was adapted from a recent study (Maccagni & Willi 2022), which was based on a universal guide (Pérez-Harguindeguy *et al.* 2013). After the first plants had started bolting, we performed leaf trait measurements. We took two leaves from the 2nd to 3rd whorl of the rosette and weighed them immediately to the nearest 0.01 mg to record the fresh weight (AT250, XA205 DualRange, Mettler Toledo, Columbus, USA). Next, we scanned leaves (CanonScan, LiDe120 and 220, Canon, Tokyo, Japan) and analysed the images using imageJ to obtain leaf area (LA). Leaves were then moved to an oven and dried at 60 °C for 72 hours (Termaks AS, Bergen, Norway), after which they were weighed again using the same balances. Leaf dry matter content (LDMC) was calculated as the ratio of dry weight over fresh weight in mg g^{-1}, and specific leaf area (SLA) as the ratio of LA over dry weight in mm^2 mg^{-1}. These values were computed and averaged across the two leaves to obtain LA, SLA and LDMC values for each plant.
3. Isotope based traits: After leaf trait measurements, we sampled fully extended leaves from the 3rd whorl for up to three replicates per family. After sampling, we froze the tissue immediately to prevent tissue degradation and further respiration. Frozen tissues were then freeze dried and shipped to the Cornell University Stable Isotope Laboratory, (Ithaca, New York, USA) where bulk leaf carbon, nitrogen and $\delta^{13}C$ were measured. We then calculated the water-use efficiency as the $\delta^{13}C$ isotopic discrimination (Farquhar *et al.* 1989) (WUE ‰) as 1000 * (-0.0085 - $\delta^{13}C$ /1000) / (1 + $\delta^{13}C$ /1000) (Seibt *et al.* 2008), where -8.5‰ was used as the $\delta^{13}C$ value for air. We obtained this

value as an average value for 2023-24 at the Germany station from the Global Monitoring Laboratory (https://gml.noaa.gov).

4. Biomass traits: Approximately one month after cumulative flowering reached a plateau, we harvested plants to measure above- and below-ground dry biomass. We took entire plants and washed them to remove soil. As roots require more rigorous cleaning to remove soil while retaining finer root material, we washed them under sieves of sizes 2mm and 0.3mm. After isolating all cleaned plant material, we divided them into four categories: the inflorescence, dead rosette, live rosette and belowground roots. We weighed them immediately to the nearest 0.1 mg to record the fresh weight (AT250, XA205 DualRange, Mettler Toledo, Columbus, USA). Plant materials were then moved to an oven and dried at 60°C for 72 h (Termaks AS, Bergen, Norway), after which they were weighed again using the same balances. Using these, we calculated the following parameters:
    a. Mass: sum of all dried plant material
    b. Aboveground biomass: sum of all aboveground dried material (excludes the belowground material).
    c. Belowground (root) biomass: dried roots
    d. Root:shoot ratio: ratio of the belowground and aboveground biomass

All traits were corrected for noise prior to analysis for the effect of block and trays with block. This was done by setting up a linear model to check if block and tray within block explained significant variation, i.e. more than 5% (reflected in the adjusted R squared values). If so, these traits were corrected for this noise. These corrected values were then used for all other analysis in this study.

S5. Model setup (additional details)

S5.1 General terms

1. "elev_Org_m": median elevation of species
2. "delta_elev": deviation of population from species' median elevation
3. "treatment": growth treatment
4. "phylo": dummy variable for species to include the phylogenetic relatedness
5. "species": species

6. “species:region:rel_elev”: the specific population
7. “genotype”: the seed family; unique identifier
8. “sigma”: the standard deviation (scale) parameter, for distributional models where the assumption of homogeneity of variances is not met (Bürkner 2017) .

Note that in “brms” syntax, “species:region:rel_elev” corresponds to the population, and is the same as writing “1 | population” where “population” uniquely identifies the sampling location.

S5.2 For LDMC, SLA, asym, WUE

Formula: trait ~ elev_Org_m * treatment + delta_elev * treatment +
(1 | gr(phylo, cov = A)) +
(1 | species) + (0 + delta_elev | species) +
(1 | species:region:rel_elev) +
(1 | genotype))

Family: Gaussian

S5.3 For leaf carbon content and growth rate

For carbon content, there were some outlier points that prevented model convergence. We therefore removed outliers beyond the 0.005 and 0.995 quartiles, which amounted to removing 10 points. For both traits, the model formula is presented below

Formula: trait ~ elev_Org_m * treatment + delta_elev * treatment +
(1 | gr(phylo, cov = A)) +
(1 | species) + (0 + delta_elev | species) +
(1 | species:region:rel_elev) +
(1 | genotype),
sigma ~ (1 | species))

Family: skew_normal

S5.4 Rest of the traits

Formula: trait ~ elev_Org_m * treatment + delta_elev * treatment +
(1 | gr(phylo, cov = A)) +
(1 | species) + (0 + delta_elev | species) +
(1 | species:region:rel_elev) +

(1 | genotype),

sigma ~ (1 | species))

Family: Gaussian

S6. Additional results

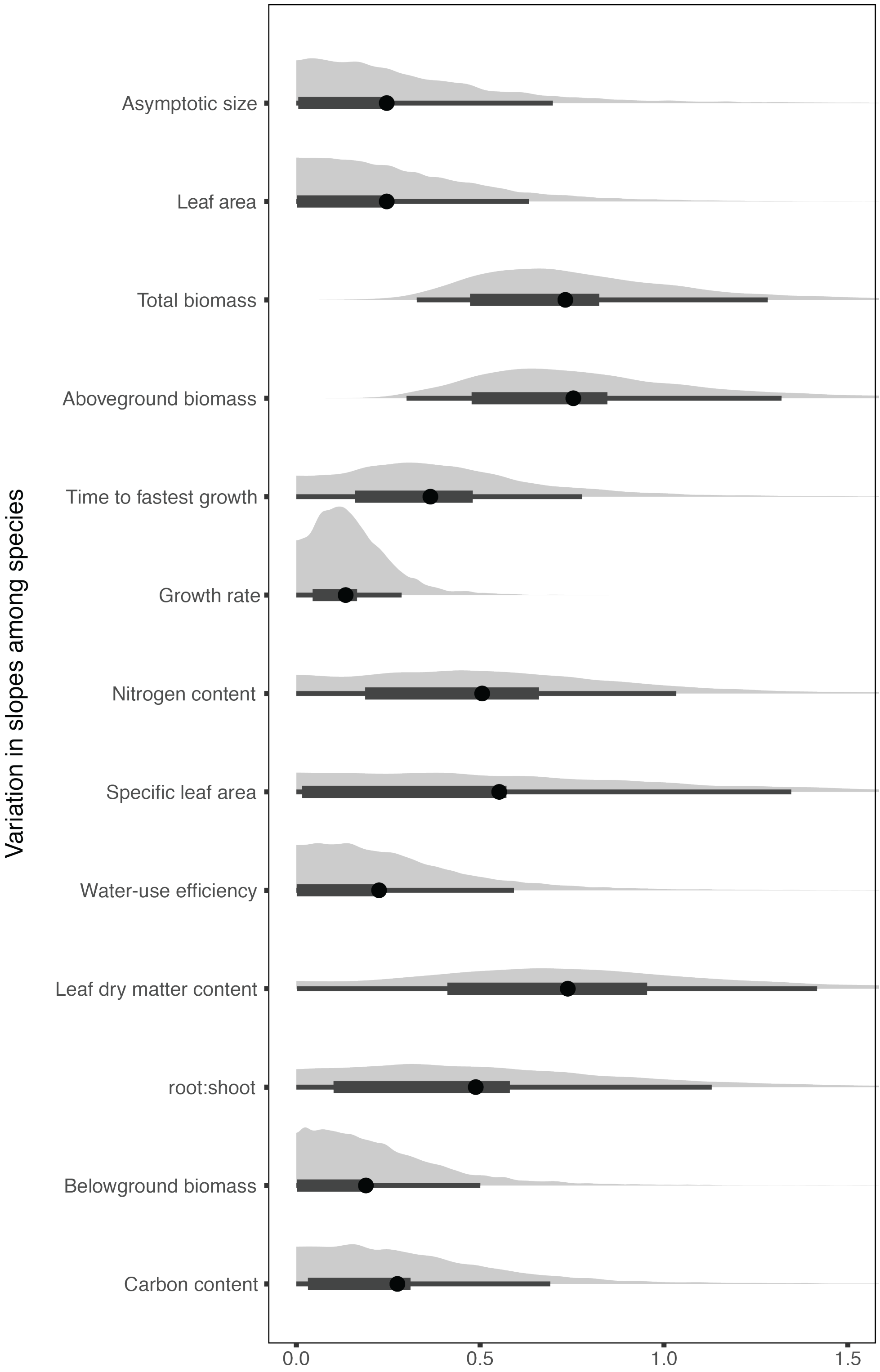

Figure S5: Variation in within-species slopes. Shown here is the distribution of standard deviation of slopes, obtained from multilevel Bayesian models. The dots depict medians with thicker and thinner lines showing the 50% and 90% highest density continuous intervals. LDMC, total biomass and aboveground biomass have larger deviations (median > 0.5), whereas for other traits, deviation in slopes is weaker (median < 0.5). Prior to analysis and

plotting, traits were rescaled to a mean of 0 and standard deviation of 1 across all species and treatments.

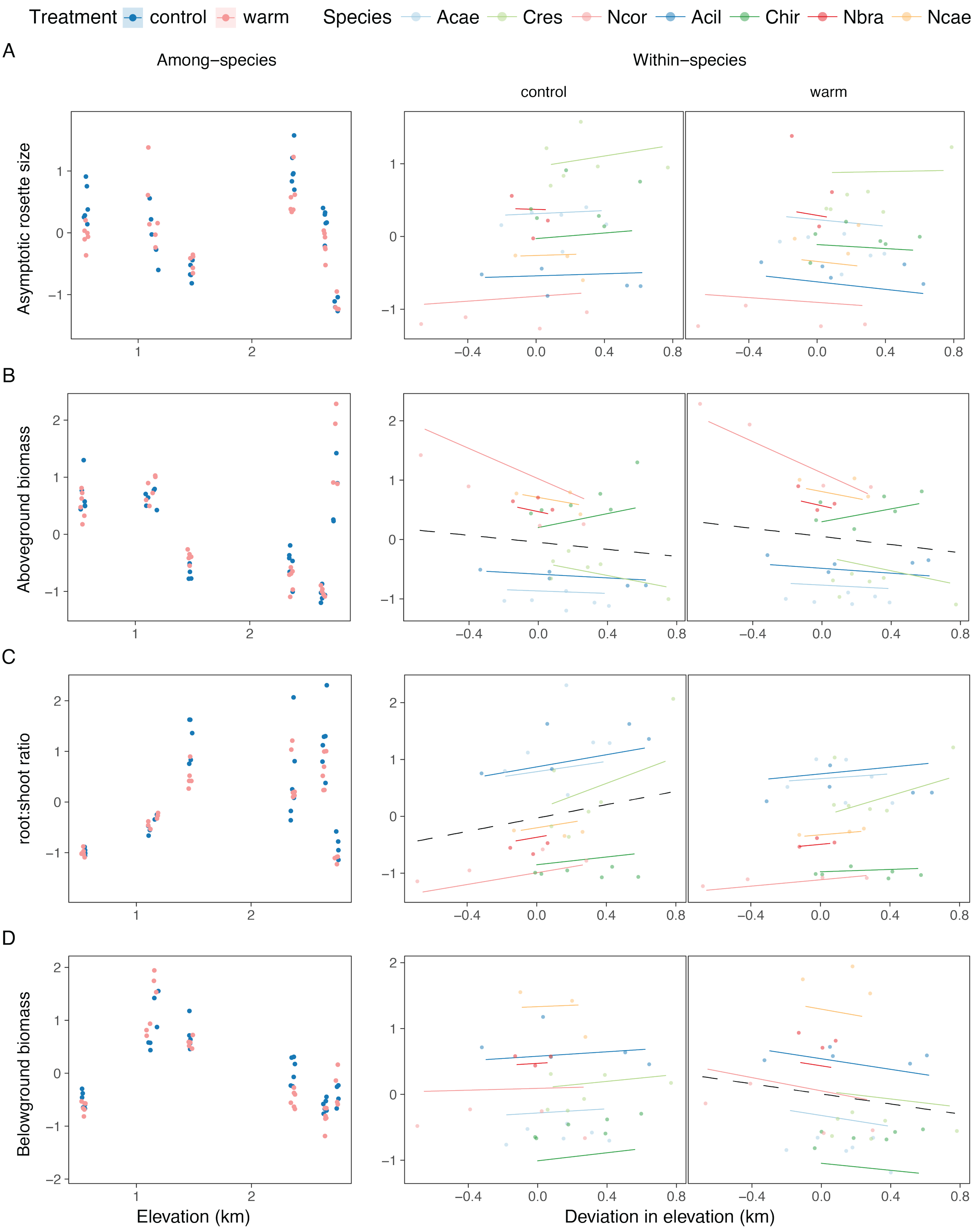

Figure S6: Trait-elevation relationships for size and allocation traits A) asymptotic size (asym) B) aboveground biomass C) root:shoot ratio and D) root biomass. Among- and within-species trait-elevation relationships are shown on the left and right panels respectively. Lines indicate

whether a general among-species (blue or pink lines) or within-species (dashed lines) relationship was found. For panels on the left, shaded regions around the slopes depict 90% highest density continuous intervals (HDCI). Panels on the right depict within-species patterns, and slopes for each species highlight the variance among within-species slopes. Points depict population-level means for each trait and were jittered slightly for better visibility. Prior to analysis and plotting, traits were rescaled to a mean of 0 and standard deviation of 1 across all species and treatments.

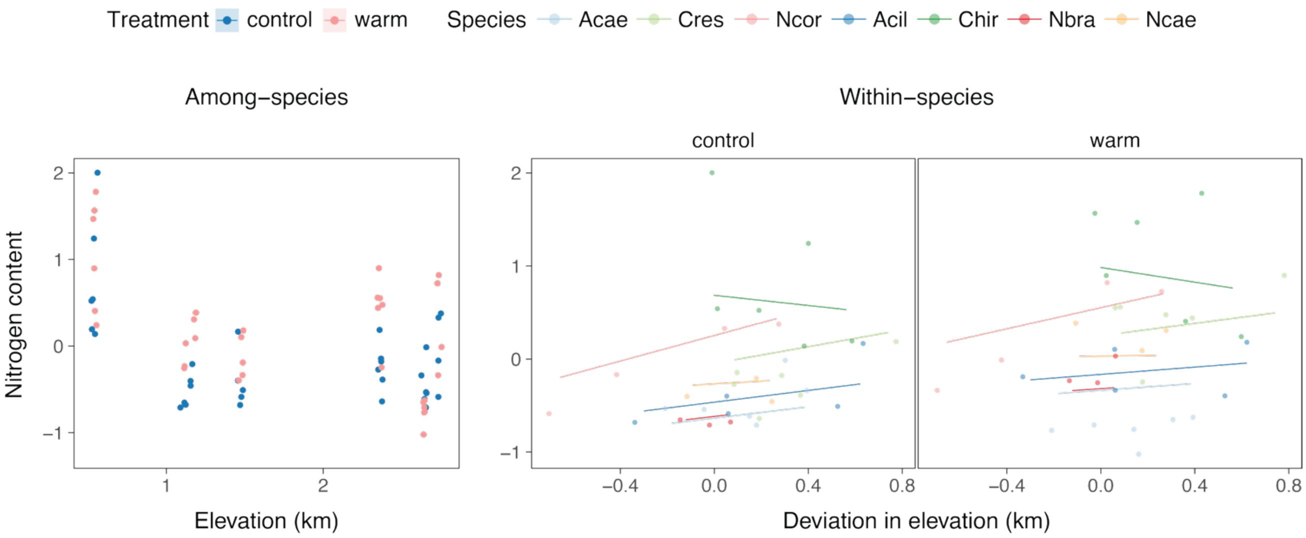

Figure S7: Trait-elevation relationships for leaf nitrogen content. Among- and within-species trait-elevation relationships are shown on the left and right panel respectively. For *N. corymbosa*, N_{mass} exhibited a change of approximately half a standard deviation across its range (brown line). Lines indicate whether a general among-species (blue or pink lines) or within-species (dashed lines) relationship was found. For the panel on the left, shaded regions around the slopes depict 90% highest density continuous intervals (HDCI). The panel on the right depict within-species patterns, and slopes for each species highlight the variance among within-species slopes. Points depict population-level means for each trait and were jittered slightly for better visibility. Prior to analysis and plotting, traits were rescaled to a mean of 0 and standard deviation of 1 across all species and treatments.

References

Bürkner, P.-C. (2017). brms : An R package for Bayesian multilevel models using Stan. *J. Stat. Softw.*, 80.

Elzhov, T.V., Mullen, K.M., Spiess, A.-N. & Bolker, B. (2023). minpack.lm: R Interface to the Levenberg-Marquardt nonlinear least-squares algorithm found in MINPACK, plus support for bounds.

Farquhar, G.D., Ehleringer, J.R. & Hubick, K.T. (1989). Carbon isotope discrimination and photosynthesis. *Annu. Rev. Plant Physiol. Plant Mol. Biol.*, 40, 503–537.

Heblack, J., Schepers, J.R. & Willi, Y. (2024). Evolutionary potential under heat and drought stress at the southern range edge of North American *Arabidopsis lyrata*. *J. Evol. Biol.*, 37, 555–565.

Maccagni, A. & Willi, Y. (2022). Trait divergence and trade-offs among Brassicaceae species differing in elevational distribution. *Evolution*, 76, 1986–2003.

Pérez-Harguindeguy, N., Díaz, S., Garnier, E., Lavorel, S., Poorter, H., Jaureguiberry, P., *et al.* (2013). New handbook for standardised measurement of plant functional traits worldwide. *Aust J Bot*, 61, 167–234.

Seibt, U., Rajabi, A., Griffiths, H. & Berry, J.A. (2008). Carbon isotopes and water use efficiency: sense and sensitivity. *Oecologia*, 155, 441.
